# Hyperthermia increases HSP production in human PDMCs by stimulating ROS formation, p38 MAPK and Akt signaling, and increasing HSF1 activity

**DOI:** 10.1186/s13287-022-02885-1

**Published:** 2022-06-03

**Authors:** Ju-Fang Liu, Po-Chun Chen, Thai-Yen Ling, Chun-Han Hou

**Affiliations:** 1 School of Oral Hygiene, College of Oral Medicine, Taipei Medical University, Taipei, 110 Taiwan; 2 Department of Life Science, National Taiwan Normal University, Taipei, 116 Taiwan; 3 Translational Medicine Center, Shin-Kong Wu Ho-Su Memorial Hospital, Taipei, 111 Taiwan; 4 Department of Medical Research, China Medical University Hospital, China Medical University, Taichung, 404 Taiwan; 5 Graduate Institute of Pharmacology, College of Medicine, National Taiwan University, Taipei, 106 Taiwan; 6 Department of Orthopedic Surgery, National Taiwan University Hospital, No. 1, Jen-Ai Road, Taipei, 100 Taiwan

**Keywords:** hPDMCs (hPDMCs), Heat shock proteins (HSPs), Heat shock

## Abstract

**Background:**

Human placenta-derived multipotent cells (hPDMCs) are isolated from a source uncomplicated by ethical issues and are ideal for therapeutic applications because of their capacity for multilineage differentiation and proven immunosuppressive properties. It is known that heat shock preconditioning induces the upregulation of heat shock proteins (HSPs), which enhance survival and engraftment of embryonic stem cells (ESCs) during transplantation in live animal models, although whether heat shock preconditioning has the same effects in hPDMCs is unclear.

**Methods:**

The hPDMCs were isolated from placenta of healthy donors. The cells were treated with heat shock (43 °C, 15 min), followed by evaluation of cell viability. Furthermore, the HSPs expression was assessed by Western blot, qPCR. The reactive oxygen species (ROS) production and signal pathway activation were determined by flow cytometry and Western blot, respectively. The regulatory pathways involved in HSPs expression were examined by pretreatment with chemical inhibitors, and siRNAs of MAPK, Akt, and heat shock factor 1 (HSF1), followed by determination of HSPs expression.

**Results:**

This study demonstrates that heat shock treatment induced ROS generation and HPSs expression in hPDMCs. Heat shock stimulation also increased p38 MAPK and Akt phosphorylation. These effects were reduced by inhibitors of ROS, p38 MAPK and Akt. Moreover, we found that heat shock treatment enhanced nuclear translocation of the HSF1 in hPDMCs, representing activation of HSF1. Pretreatment of hPDMCs with ROS scavengers, SB203580 and Akt inhibitors also reduced the translocation of HSF1 induced by heat shock.

**Conclusions:**

Our data indicate that heat shock acts via ROS to activate p38 MAPK and Akt signaling, which subsequently activates HSF1, leading to HSP activation and contributing to the protective role of hPDMCs.

## Introduction

The ability of stem cells to self-renew and differentiate into multiple cell lineages and tissue types is crucial to regenerative medicine, where clinical investigations are seeking effective ways to repair, replace and regenerate cells, tissues and organs that are damaged, injured or diseased. Various biomedical approaches in regenerative medicine involve the use of stem cells, but current invasive procedures and ethical issues limit this therapeutic source, such as the isolation of stem cells from the human embryo [1, 2]. Alternative sources of human stem cells are needed that are safe, easily accessible and provide high cell yields.

It is suggested that umbilical cord blood, amniotic fluid and placental tissue are easily procurable sources of stem cells that do not involve the need for invasive procedures [3, 4]. Regenerative medicine investigations have used human placenta-derived multipotent cells (hPDMCs) as an useful replacement for embryonic stem cells (ESCs), in recognition of the fact that hPDMCs are capable of self-renewal, of differentiating into a variety of differentiated cells, and they possess immunomodulatory properties [5]. Moreover, not only are hPDMCs essential in embryonic and fetal development, but they can differentiate into multiple adult cell types and therefore hold enormous potential for stem cell therapy [6]. Indeed, hPDMSCs are considered to behave as young progenitor cells and they have a reputation of overcoming immunological barriers involving host immune responses in allogeneic transplantation [7, 8].

Heat shock proteins (HSPs) help to maintain cellular homeostasis and are upregulated under stressful physiological conditions, when they play a crucial role by refolding proteins into their correct structure and assist with protein assembly [9, 10]. Heat shock preconditioning induces the upregulation of HSPs, which enhance survival and engraftment of ESCs during transplantation in live animal models [11–14]. Human ESCs are more capable than differentiated cells at resisting oxidative stress and irradiation, which may be because of the upregulated levels of HSP expression in response to stressors [15]. This is supported by the finding that HSP overexpression enhances cell survival and engraftment during transplantation [14, 16]. A range of stressors, such as heat, anoxia, ischemia, heavy metal ions, ethanol, nicotine, surgical stress and viral agents, can activate the binding of the transcription factor heat shock factor-1 (HSF1) to the heat shock element (HSE) and upregulate levels of HSP expression, including HSP27, HSP70, and HSP90 [17, 18]. Evidence suggests that HSF1-induced mediation of HSP expression following hyperthermia and heat shock protects cells from the adverse consequences of stress [19]. Heat shock treatment induces the production of reactive oxygen species (ROS), triggering the activation of an intercellular signal transduction pathway [20–22]. ROS are crucial for cell growth, migration, differentiation and apoptosis, and are generated by various sources including stress, nicotinamide adenine dinucleotide phosphate (NADPH) oxidase and heat [20–23]. Importantly, ROS can act as signaling molecules for the activation of various signaling pathways [24] and heat shock induces ROS and HSP production by activating Akt and p38 MAPK signaling pathways in human esophageal microvascular endothelial cells (HEMECs) [20].

Little is known about the effects of heat shock treatment on hPDMCs. We therefore used hPDMCs to investigate the involvement of heat shock signaling pathways in HSP production. Our results show that heat activates the ROS/NADPH, p38 MAPK, Akt and HSF pathways, subsequently upregulating HSP production. These data help to elucidate the role of HSPs in modulating the survival of hPDMCs.

## Materials and methods

### Materials

Protein A/G PLUS-Agarose, anti-rabbit IgG-HRP secondary antibody, rabbit polyclonal antibodies against HSP90, HSP70, HSP27, p38 MAPK, JNK, ERK, Akt, HSF1 and β-actin, small interfering RNAs (siRNAs) against HSP90, HSP70 and HSP27 were obtained from Santa Cruz Biotechnology (Santa Cruz, CA, USA). The following rabbit polyclonal antibodies, including phospho-p38 MAPK (Thr180/Tyr182), phospho-SAPK/JNK (Thr183/Tyr185), phospho-p44/42 MAPK (Erk1/2) (Thr202/Tyr204), and phospho-Akt (Ser473), were obtained from Cell Signaling and Neuroscience (Danvers, MA, USA). All other chemicals were purchased from Sigma-Aldrich (St. Louis, MO, USA).

### Cell cultures

hPDMCs were isolated using enzyme treatment from term placentas (at 38–40 weeks’ gestation) obtained with informed consent from healthy donor mothers, following approval granted by the Institutional Review Board of Taipei Medical University (201005015). Briefly, the placental tissue was minced into small pieces, enzymatic digestion, then maintained in complete medium consisting of Dulbecco's Modified Eagle Medium (DMEM) with 20 mM HEPES and 10% Fetal bovine serum (FBS), 100 U/ml penicillin, 100 µg/ml streptomycin, and 2 mM glutamine. All cell culture materials were purchased from Invitrogen (Carlsbad, CA, USA).

The cells were maintained in incubator provide with humidified 5% CO_2_ atmosphere at 37 °C. The medium was renewed once or twice weekly. hPDMCs between the passages of 5 to 8 were used in all experiments [25]. The cell maintained in complete media was subjected to heat shock treatment in an oven at 43 °C for 15 min. For post-treatment recovery, the cells were maintained in an incubator at 37 °C until required for analysis [18].

### Assessment of cell viability

3-[4, 5-dimethylthiazol-2-yl]-2,5-diphenyltetrazolium bromide (MTT) was used for assessment of cell viability. The cells treated with heat shock were performed with MTT assay after 48 h post-treatment. The MTT (0.5 mg/ml) was added in wells and incubated for 2 h at 37 °C. Then, the dimethyl sulfoxide (DMSO) was added to dissolve formazan crystals. The absorbance at 550 nm (reference wavelength 650 nm) was measured using a microplate reader (Bio-Tek, Winooski, VT, USA).

### Quantitative polymerase chain reaction (qPCR)

The hPDMCs grown in 6-well plates were treated with heat shock, followed by collecting total RNA using TRIzol kit (MDBio Inc., Taipei, Taiwan). The 2 µg RNA were subjected to reverse transcription to produce cDNA templates. qPCR was carried out using SYBR Green (KAPA Biosystems). The reactions were performed in triplicate using StepOnePlus (Applied Biosystems, Foster City, CA, USA) according to the manufacturer’s instruction. The PCR probes used in qPCR reaction were obtained from Applied Biosystems. The expression level of target gene was normalized by the comparative threshold cycle (ΔΔCt) method. All the primers used in qPCR are list as follows:

Human HSP90, forward primer: 5’-AGAGCCTACGTTCCTGCACT-3’ and reverse primer: 5’-GACCATTCTTCTAGAGCATTCAGG-3’;

Human HSP70, forward primer: 5’-TAACCCCATCATCAGCGGAC -3’ and reverse primer: 5’-GAAGCTCCAAAACAAAAACAGCA-3’;

Human HSP27, forward primer: 5’-ACATTTGCTCGGTCACTCC-3’ and reverse primer: 5’-AAGCCGTGCTCATCTTGTCT-3’;

Human GADPH, forward primer: 5’-ACCCACTCCTCCACCTTTG -5’ and reverse primer:5’- CTGTAGCCAAATTCGTTGTCAT -3’.

### Detection of reactive oxygen species (ROS)

Intracellular ROS were detected by using dihydrorhodamine 123 (DHR123). The cells (5 × 10^5^) grown in 24-well plates were treated with heat shock. The cells were incubated with DHR123 (20 μM) for 10 min at 37 °C and cellular fluorescence intensity was detected using a flow cytometry analysis at 488 nm.

### Western blot analysis

The cells grown in 6-well plates were treated with heat shock, followed by collecting cell lysates using RIPA lysis buffer. The 20 μg cell lysates were separated using 12% SDS-PAGE and then transferred to PVDF membranes. The membranes were incubated with 4% BSA solution for 1 h at room temperature. Subsequently, the membranes were incubated with primary antibodies (HSP90, HSP70, HSP27, p38 MAPK, JNK, ERK, Akt, HSF1 and β-actin) with 1:1000 dilution for 1 h at room temperature. After 3 washed with TBST, the membranes were incubated secondary antibody with 1:3000 dilution for 1 h at room temperature. Finally, the membranes were incubated ECL reagents (Amersham Pharmacia Biotech, Inc, USA) and the signals were detected with Kodak X-OMAT LS film (Eastman Kodak, Rochester, NY, USA).

### siRNA transfection

siRNAs which specific for human HSP27, HSP70 and HSP90, and control siRNA, were obtained from Santa Cruz Biotechnology. Cells were transfected with 100 nM siRNAs using Lipofectamine 2000 (Invitrogen Life Technology, Waltham, MA, USA), according to the manufacturer's instructions [26].

### Immunofluorescence staining

Cells grown in 12 mm coverslips were treated with heat shock and fixed with 4% paraformaldehyde at room temperature for 30 min. After washing with PBS for 3 times, the cells were permeabilized with 4% nonfat milk in PBS containing 0.5% Triton X-100. The cells were incubated with primary antibody (HSF-1) with 1:100 dilution and FITC-conjugated secondary antibody with 1:500 dilution for 1 h. Finally, the fluorescence was monitored under a Zeiss fluorescence microscope [27].

### Statistical analysis

All values reported are means ± SEM. Comparisons between two samples were performed using the Student’s *t *test. Comparisons between more than two samples were performed using one-way analysis of variance (ANOVA), followed by the Bonferroni post hoc test for multiple comparisons. In all cases, differences were deemed significant when *p* < 0.05.

## Results

### Heat shock treatment induces HSP production in PDMCs

**Fig. 1:**
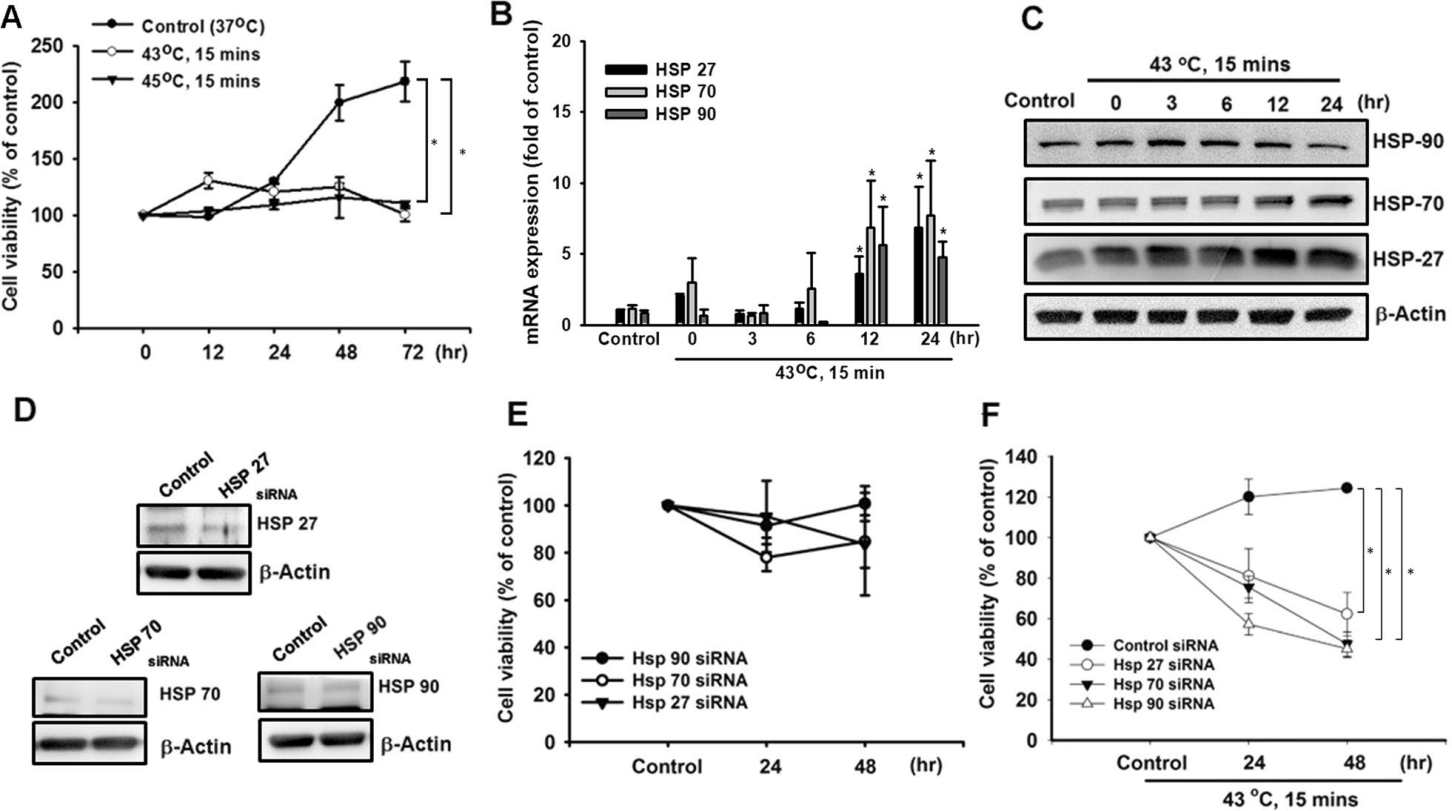
The effects of heat shock on cell viability and HSP production in PDMCs. **A** PDMCs were incubated with heat shock for 15 min, followed by determining cell viability by MTT assay after 48 h. **B**, **C** PDMCs were incubated with heat shock for 15 min; the total RNA and protein were collected after 0, 3, 6, 12, and 24 h. The expression levels of HSPs were determined by qPCR and Western blotting. **D** The HSPs siRNA or control siRNA were introduced into PDMCs for 24 h, follow by determining the protein expressions by Western blotting. **E** The PDMCs were treated as Fig. 1D described, follow by determining cell viability by MTT assay. **F** The HSPs siRNA or control siRNA were introduced into PDMCs for 24 h. Subsequently, the cells were treated with heat shock for 15 min. Forty-eight hours later, the cell viability was determined by MTT assay. Results are expressed as the mean ± SEM. *p < 0.05 compared with control

Heat shock pretreatment is beneficial for the survival and engraftment of transplanted cells [12–14]. We therefore used hPDMCs to investigate the involvement of heat signaling pathways in HSP production. Initial heat treatment of PDMCs did not affect cell viability (Fig. 1A). We examined levels of HSP expression in hPDMCs with Western blot and qPCR analysis. The PDMCs were treated with heat shock (43 °C) for 15 min, followed by determining the HSP production after 24 h post-treatment. The results indicated that heat shock treatment time-dependently induced HSP production (Fig. 1B, C). PDMCs were then transfected for 24 h with HSP90, HSP70 and HSP27, which inhibited HSP expression (Fig. 1D) and had no significant effect on cell viability (Fig. 1E). However, transfection with siRNAs of HSPs decreased the protective effects of HSPs upon cell survival after heat shock treatment (Fig. 1F). These data indicated that heat increases HSP production in human PDMCs and protects cell survival.

### ROS and NADPH are involved in the heat-induced production of HSPs in hPDMCs

**Fig. 2:**
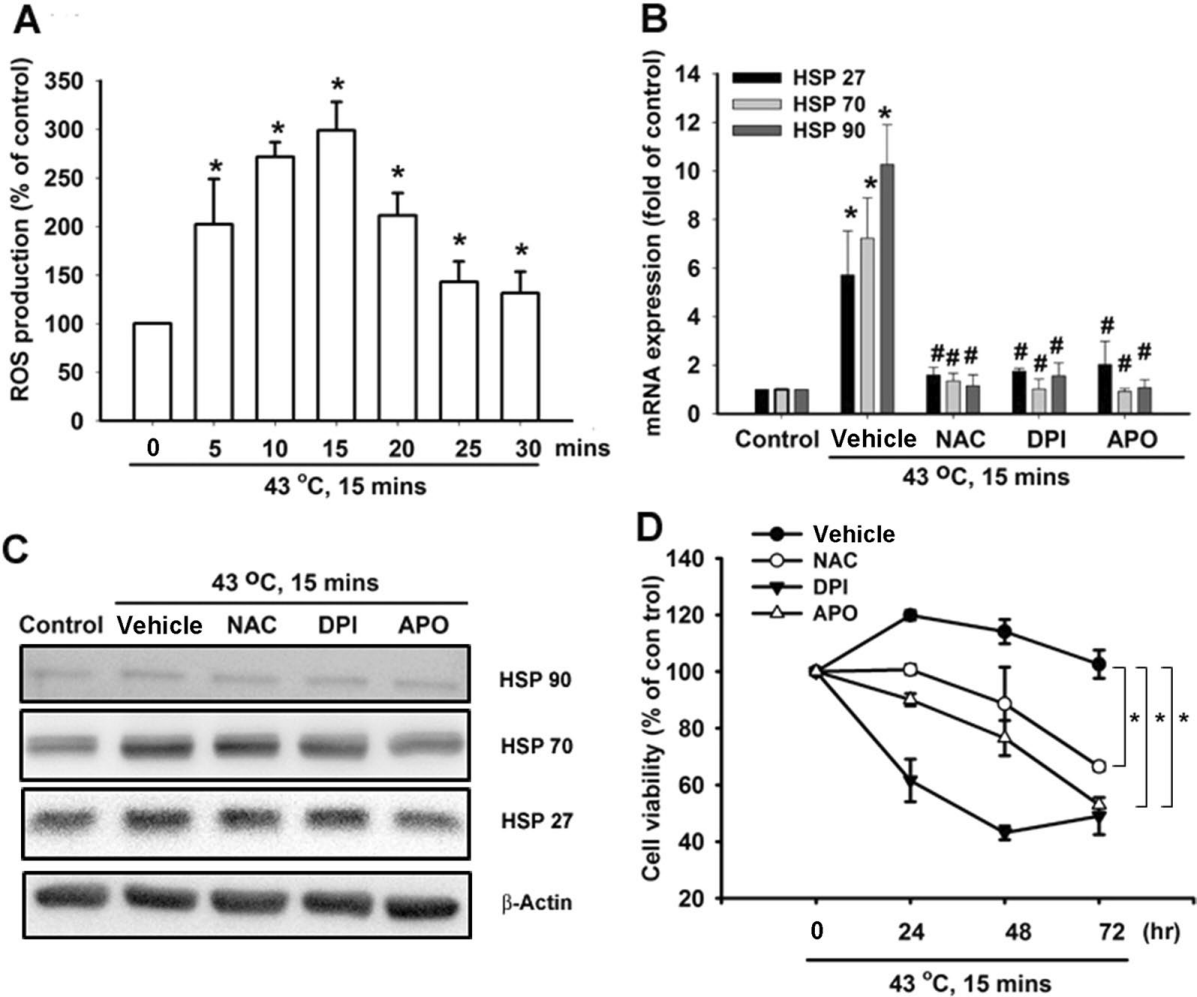
Heat shock-induced ROS production in PDMCs. **A** PDMCs were incubated with heat shock, and the flow cytometry was conducted to examine ROS production in different time points. **B**, **C** PDMCs were pretreated with DMSO vehicle, NAC (5 µM), DPI (5 µM) and APO (5 µM) for 30 min, and then stimulated with heat shock. The cells were collected at 48 h after the heat shock, and the mRNA and the protein expression of HSPs were examined by qPCR and Western blotting. **D** PDMCs were treated as described in Fig. 2B, and then, the cell viability was determined at different time points by MTT assay. Results are expressed as the mean ± SEM. *p < 0.05 compared with control; #, *p* < 0.05 compared with heat shock -treated group

ROS production is triggered by different stressors such as oxidative stress, heavy metal stress and hyperthermia [22], as well as heat shock [20, 21]. Elevations in levels of superoxide anions (O_2_^−^), hydrogen peroxide (H_2_O_2_) and nitric oxide (NO) are observed in multiple cell lines and tumor tissues subjected to heat shock [28, 29]. When we sought to determine the involvement of ROS accumulation in heat shock-induced HSP production in PDMCs, DHR123-based FACS detection demonstrated increased levels of cellular ROS in PDMCs treated with heat shock (Fig. 2A). Pretreatment of cells with N-acetylcysteine (NAC), a direct scavenger of ROS, reduced the increase in HSP production induced by heat shock (Fig. 2B-C). NADPH oxidase is an important enzymatic source for the production of ROS under pathologic conditions [23], so we investigated the role of NADPH oxidase in heat shock-induced HSP production in hPDMCs. Pretreatment of cells with NADPH oxidase inhibitors (DPI and APO) inhibited HSP production induced by heat shock (Fig. 2B,C). In order to investigate whether heat shock-induced HSP production via ROS protects the survival of hPDMCs, we treated cells with ROS and NADPH oxidase inhibitors and found that these antagonized the protection conferred by heat shock upon hPDMC survival (Fig. 2D). These data implicate the involvement of ROS and NADPH oxidase activation in heat shock-induced HSP production in hPDMCs.

### p38 MAPK and Akt are involved in heat-induced production of HSPs in hPDMCs

**Fig. 3:**
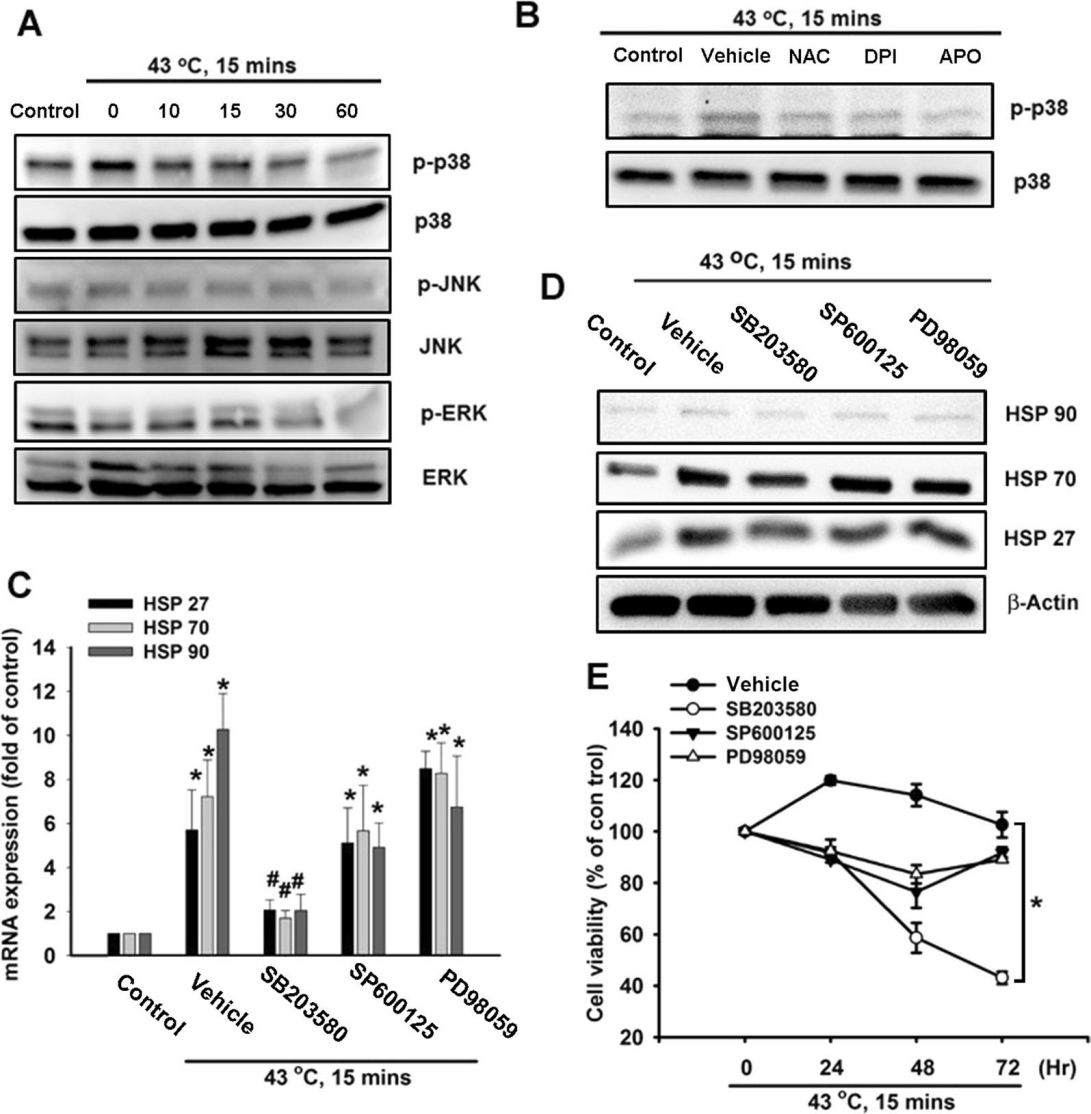
p38 MAPK is involved in heat shock-mediated HSPs production in PDMCs. **A** PDMCs were treated with heat shock, and p38 MAPK, JNK and ERK phosphorylation were assessed at different time points by Western blotting. **B** PDMCs were treated with DMSO vehicle, NAC (5 µM), DPI (5 µM) and APO (5 µM) for 30 min, followed by heat shock treatment. The treated cells were collected 15 min later, and p38 MAPK phosphorylation was determined by Western blotting. **C**, **D** PDMCs were pretreated with DMSO vehicle, SB203580 (10 µM), SP600125 (10 µM) and PD98059 (10 µM) for 30 min, followed by heat shock treatment. The treated cells were collected 24 h later, and gene expression of HSPs was assessed by qPCR and Western blotting. (E) PMDCs were pre-incubated with DMSO vehicle, SB203580 (10 µM), SP600125 (10 µM) and PD98059 (10 µM) for 30 min, followed by heat shock treatment. The cell viability was determined at different time points by MTT assay. Results are expressed as the mean ± SEM. *p < 0.05 compared with control; #, *p* < 0.05 compared with heat shock-treated group

**Fig. 4:**
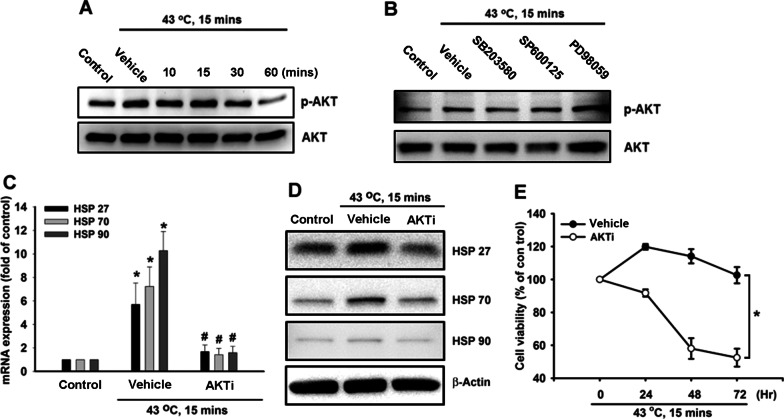
Akt is responsible for HSP production after heat shock treatment. **A** PDMCs were treated with heat shock, and Akt phosphorylation was assessed at different time points by Western blotting. **B** PDMCs were pretreated with DMSO vehicle, SB203580 (10 µM), SP600125 (10 µM) and PD98059 (10 µM) for 30 min, followed by heat shock treatment, and Akt phosphorylation were examined by Western blotting. **C**, **D** PDMCs were pretreated with DMSO vehicle and Akti (10 µM) for 30 min, followed by heat shock treatment. The treated cells were collected 24 h later, and gene expression of HSPs was assessed by qPCR and Western blotting. **E** PDMCs were pretreated with DMSO vehicle and Akti (10 µM) for 30 min, followed by heat shock treatment, and the cell viability was evaluated at different time points by MTT assay. Results are expressed as the mean ± SEM. *, *p* < 0.05 compared with control; #, *p* < 0.05 compared with heat shock-treated group

Several reports have revealed that activation of Akt and p38 MAPK signal pathways was required for heat shock response in different cell types [20, 30, 31]. Therefore, we suggested that Akt and p38 signal proteins were the major components involved in heat shock response in stem cells as well, which was poorly described before. We then measured p38 MAPK phosphorylation in hPDMCs in response to heat shock and observed time-dependent phosphorylation of p38 MAPK, but not JNK or ERK (Fig. 3A). NAC, DPI and APO treatment all attenuated the increase in p38 MAPK phosphorylation induced by heat shock (Fig. 3B). Thirty minutes of pretreatment with a p38 MAPK inhibitor (SB203580, 10 µM) markedly inhibited heat shock-induced HSPs mRNA expression (Fig. 3C); no such effects were observed after pretreating the cells for 30 min with either a JNK inhibitor (SP600125, 10 µM) or ERK inhibitor (PD98059, 10 µM). Moreover, pretreating the PDMCs with the p38 MAPK inhibitor (SB203580) reduced levels of heat shock-induced protein expressions of HSPs, while the JNK inhibitor (SP600125) and ERK inhibitor (PD98059) had no such effects (Fig. 3D). In order to confirm whether heat shock-induced HSP production via p38 MAPK protects the survival of hPDMCs, we treated cells with the p38 MAPK inhibitor and found that this antagonized the protective effects on hPDMCs induced by heat shock, whereas JNK and ERK inhibitors had no such effects (Fig. 3E). Akt phosphorylation on Ser^473^ by a p38 MAPK-dependent signaling pathway causes enzymatic activation [32]; therefore, we evaluated Akt activation in hPDMCs after heat shock treatment. The result indicated that Akt phosphorylation was increased after heat shock (Fig. 4A). Treatment with p38 inhibitor but not JNK or ERK inhibitors obviously inhibited Akt phosphorylation (Fig. 4B), suggesting heat shock-induced Akt activation was regulated by p38. We further pretreated hPDMCs with the Akt inhibitor and found that antagonized heat shock-induced HSP production (Fig. 4C-D). Finally, pretreatment of Akt inhibitor also abolished protective effects induced by heat shock in hPDMCs. In summary, heat shock appears to act via the p38 MAPK and AKT-dependent signaling pathways and thereby enhances HSP production in PDMCs.

### The p38 MAPK and Akt signaling pathway involves HSF1 in heat shock-induced HSP production

**Fig. 5:**
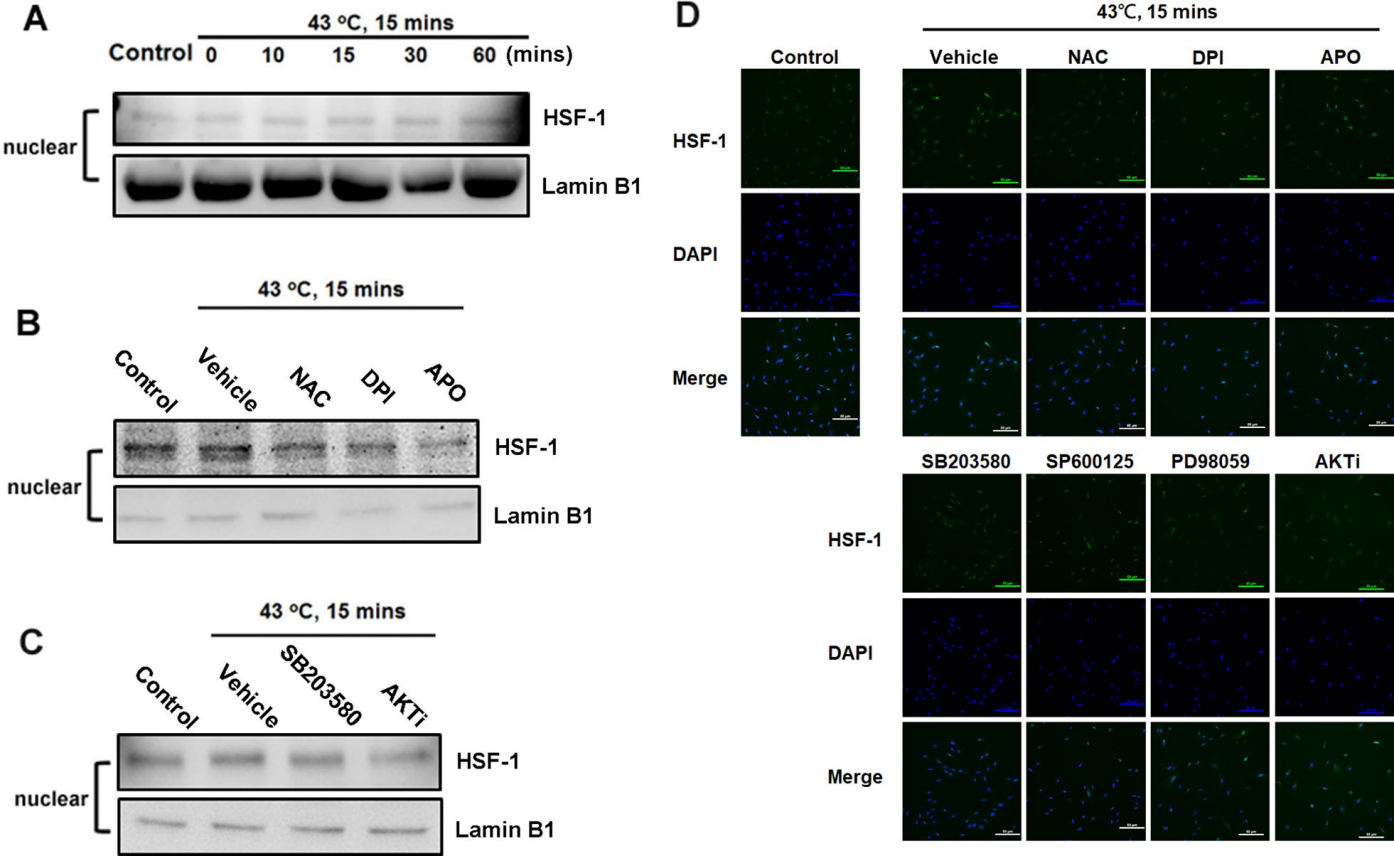
Heat shock-induced HSF1 activates through ROS, p38 MAPK/Akt pathway. **A** PDMCs were incubated with heat shock for indicated time intervals, and HSF1 expression in cytosol and nucleus was determined by Western blotting. **B** PDMCs were pretreated for 30 min with DMSO vehicle, NAC (5 µM), DPI (5 µM) and APO (5 µM) followed by stimulation with heat shock (43 °C, 15 min) for 60 min, and HSF1 expression in nucleus was determined by Western blotting. **C** PDMCs were pretreated for 30 min with DMSO vehicle, SB203580 (10 µM) and Akti (10 µM) followed by stimulation with heat shock (43 °C, 15 min) for 60 min, and HSF1 expression in nucleus was determined by Western blotting. **D** PDMCs were pretreated with DMSO vehicle, NAC, DPI, APO, SB203580, SP600125, PD98059 and Akti for 30 min followed by stimulation with heat shock for 60 min, and HSF1 immunofluorescence staining was examined (Scale bar = 50 μm). Results are expressed as the mean ± SEM. *p < 0.05 compared with control; #*p* < 0.05 compared with heat-treated group

Many types of stressors activate the binding of HSF1 to HSE elements and induce HSP expression (17, 18). The HSF1 pathway is therefore important in heat shock-induced HSP production in hPDMCs. Moreover, when subjected to stress, the HSF1 protein translocates from the cytosol to the cell nucleus [33]. We observed that heat shock enhanced HSF1 translocation from the cytosol to the cell nucleus (Fig. 5A). Similarly, pretreating hPDMCs with NAC, DPI, APO, SB203580 and Akt inhibitors reduced the heat shock-induced translocation of HSF1 into the nucleus (Fig. 5B–D). Our findings indicate that the heat shock-induced increase in HSP expression in PDMCs requires activation of the ROS, p38 MAPK, AKT and HSF1 pathways.

## Discussion

The isolation of hPDMCs from placental tissue is not complicated by ethical issues [8]. Such cells are capable of self-renewal, of multipotent differentiation and they possess immunomodulatory properties, making them desirable as an alternative source of ESCs in regenerative medicine [5]. Other advantages of hPDMCs include their ability to behave similarly to young progenitor cells and their capacity to overcome immunological barriers involving host immune responses in allogeneic transplantation [7, 8].

Damaged and diseased organs produce free radicals, inflammatory cytokines, toxins and active apoptotic signals, all of which reduce the efficacy of transplanted cells [34, 35]. Heat shock pretreatment can promote the survival of transplanted cells by inducing chaperone-mediated proteins [36, 37], including HSPs and nuclear/histone chaperones, which maintain protein folding homeostasis under both normal and stressful physiological conditions [9, 38]. Fever, nutritional deficiency, inflammation, oxidative stress and viral infection can activate the binding of HSF1 to HSE elements and induce HSP27, HSP70 and HSP90 expression (17, 18). HSPs are housekeeping proteins that are important for cell survival and show superiority over various differentiated cells in their capacity for self-renewal, differentiation and high stress tolerance [39, 40]. In this study, we found that heat shock (at 43 °C for 15 min) treatment increased HSP expression without apparently affecting the viability of hPDMCs. Although 45 °C for 15 min heat shock in PDMCs did not reduce cell viability as well, we have checked with previous studies, which indicated moderate/mild hyperthermia (i.e., heating at ≤ 43 °C) could induce thermotolerance by HSPs expression in cells [41]. However, at the temperature above 43 °C, the HSPs expression is diminished as well as its folding capacity [42, 43]. The unfold protein response is elevated and results in cell death. Our results here suggest that mesenchymal stem cells may have higher thermotolerance, but more investigations are needed to determine whether this is the case.

HSPs are proposed to modulate cell differentiation through various mechanisms. For instance, HSP27 was proposed as differentiation marker of skin keratinocytes and muscle cells [44, 45]. Moreover, several reports have indicated that HSP70 exhibited critical role in erythroblasts differentiation. HSP70 has been shown to bind to GATA-1 [46] or Apoptosis-inducing factor (AIF) [47], therefore protecting cell from apoptosis and allowing the differentiation of erythroblasts. HSP90 was also implicated in embryonic development and cell differentiation in different species, such as *Drosophila melanogaster* [48], *Caenorhabditis elegans* [49], zebrafish [50], and mice [51]. Here, we found these HSPs were increased after heat shock in PDMCs. These results suggest heat shock could regulate differentiation in PDMCs.

We found that heat shock elevated levels of HSP mRNA and protein expression via ROS induction of p38 and Akt signaling, which regulates the survival of hPDMCs. Our evidence is the first to show that heat shock treatment of hPDMCs induces increases in HSP expression and is protective of cell survival. These data indicate that the heat shock pathway is a mediator in the survival of hPDMCs.

Heat shock treatment has previously been shown to induce ROS production, which activates the intercellular signal transduction pathway [52]. In this study, we demonstrated that ROS production was required for heat shock-induced HSP production. Treating hPDMCs with a ROS inhibitor reduced heat shock-induced HSP production and inhibited the heat shock-mediated protection of hPDMC survival. Thus, it appears that ROS is involved in heat shock-induced HSP production and HSP release from hPDMCs.

Researchers have demonstrated that heat shock induces ROS and HSP production by activating Akt and p38 MAPK in HEMECs [20]. Our study found that heat shock enhanced p38 and Akt phosphorylation in hPDMCs and that heat shock-induced production of HSPs was effectively inhibited by treatment with a p38 or Akt inhibitor. Furthermore, treatment with a ROS inhibitor and a NADPH inhibitor blocked p38 activation induced by heat shock. These results were further confirmed when we observed that heat shock-induced protection of hPDMC survival was inhibited by a p38 or Akt inhibitor. Our data suggest that the p38/Akt pathway plays an important role in levels of HSP expression in PDMCs.

HSF1 has been reported as a major regulator in mediating expression of HSPs in response to heat shock. The signal pathways responsible for HSF1 activation are also summarized in previous article [53]. We found in this study that heat shock increased levels of HSF1 expression in the cell nucleus and that HSF1 activation contributed to heat shock-induced HSP production in hPDMCs. Thus, HSF1 is important in heat shock-induced HSP production. We also observed that treating hPDMCs with ROS, NADPH, p38 MAPK and Akt inhibitors reduced heat shock-induced HSF1 activity. It appears that the interactions between heat shock and these signaling molecules, as well as ROS, upregulate HSP expression via p38, Akt and HSF1 signaling in hPDMCs.

## Conclusion

**Fig. 6:**
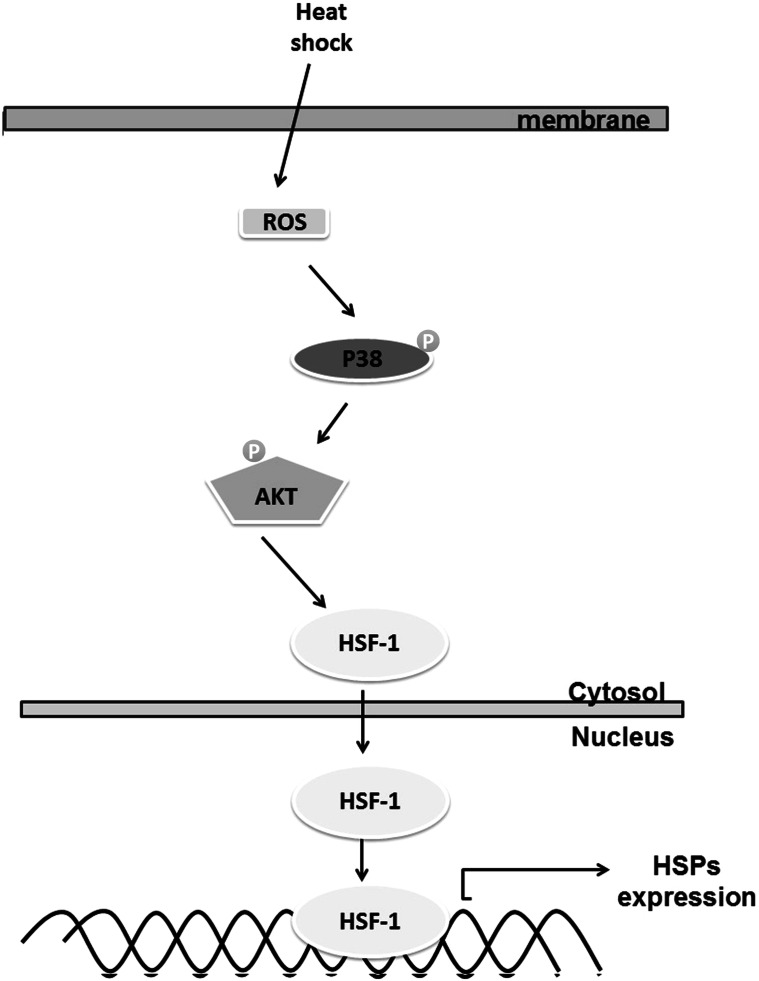
Schematic diagram of the signaling pathways involved in heat shock-induced HSPs expression in PDMCs. Heat shock increases HSPs expression by ROS and activating p38 and Akt, which enhances HSF1 expression in nucleus. This result indicates the transactivation of HSPs expression

Our investigations into the signaling pathways involved in heat shock-induced HSP production in hPDMCs demonstrate that heat shock increases HSP production by inducing ROS expression and activating p38/Akt signaling, thereby enhancing HSF1 transcription activity which contributing to HSP production (Fig. 6). This evidence of a heat shock-mediated signaling pathway improves our understanding of the mechanisms underlying the survival of hPDMCs under hyperthermic conditions. This discovery could help to inform the development of more effective therapies in regenerative medicine.

## Data Availability

The datasets used and/or analyzed during the current study are available from the corresponding author on reasonable request.

## Abbreviations

hPDMCs: Human placenta-derived multipotent cells
HSPs: Heat shock proteins
ESCs: Embryonic stem cells
ROS: Reactive oxygen species
HSF1: Heat shock factor 1
HEMECs: Human esophageal microvascular endothelial cells
siRNAs: Small interfering RNAs
qPCR: Quantitative polymerase chain reaction
NAC: N-acetylcysteine
MTT: 3-(4,5-Dimethylthiazol-2-yl)-2,5- diphenyltetrazolium bromide
DHR123: Dihydrorhodamine 123
ANOVA: One-way analysis of variance
